# Cardiovascular outcomes associated with crush versus provisional stenting techniques for bifurcation lesions: a systematic review and meta-analysis

**DOI:** 10.1186/s12872-019-1070-y

**Published:** 2019-04-23

**Authors:** Feng Huang, Zu-chun Luo

**Affiliations:** 1 Institute of Cardiovascular Diseases and Guangxi Key Laboratory Base of Precision Medicine in Cardio-cerebrovascular Disease Control and Prevention and Guangxi Clinical Research Center for Cardio-cerebrovascular Diseases, the First Affiliated Hospital of Guangxi Medical University, Nanning, 530021 Guangxi China; 2 Department of Internal Medicine Education, the First Affiliated Hospital of Guangxi Medical University, Nanning, 530021 Guangxi China

**Keywords:** Percutaneous coronary intervention, Crush stenting technique, Provisional stenting technique, Major adverse cardiac events, Repeated revascularization, Coronary bifurcation lesions

## Abstract

**Background:**

Percutaneous coronary intervention (PCI) for bifurcation lesions has often been challenging for Interventionists. Application of the correct intra-procedural technique is vital to generate beneficial outcomes after PCI. We aimed to systematically compare the post interventional cardiovascular outcomes which were reported using crush versus provisional stenting techniques for bifurcation lesions.

**Methods:**

A computerized search was carried out through Medical Literature Analysis and Retrieval System Online, EMBASE, the Cochrane Central and through www.ClinicalTrials.gov for English publications comparing crush versus the provisional stenting techniques for coronary bifurcation lesions during PCI. Major adverse cardiac events, all-cause mortality, cardiac death, myocardial infarction, stent thrombosis, target vessel and target lesion revascularizations were the endpoints in this analysis. Odds ratios (OR) and 95% confidence intervals (CI) were generated during statistical analysis to represent the data.

**Results:**

Six studies consisting of a total number of 2220 participants (1085 participants were assigned to the crush stenting technique and 1135 participants were assigned to the provisional stenting technique) enrolled between years 2004 and 2016 were included in this analysis.

During a follow-up time period from six to sixty months, major adverse cardiac events (OR: 0.73, 95% CI: 0.59–0.91; *P* = 0.005), target vessel revascularization (OR: 0.62, 95% CI: 0.43–0.89; *P* = 0.01) and target lesion revascularization (OR: 0.62, 95% CI: 0.45–0.85; *P* = 0.003) were significantly lower in patients who were assigned to the crush stenting technique. However, all-cause mortality (OR: 0.90, 95% CI: 0.48–1.68; *P* = 0.74), cardiac death (OR: 0.56, 95% CI: 0.29–1.08; *P* = 0.08), myocardial infarction (OR: 0.89, 95% CI: 0.62–1.27; *P* = 0.53) and stent thrombosis (OR: 0.72, 95% CI: 0.36–1.42; *P* = 0.34) were not significantly different.

**Conclusion:**

In patients with coronary bifurcation lesions undergoing PCI, crush stenting technique was associated with significantly lower major adverse cardiac events and repeated revascularization without any change in mortality, myocardial infarction and stent thrombosis when compared to the provisional technique showing a benefit of crush over the provisional stenting technique during PCI.

## Background

Percutaneous coronary intervention (PCI) for bifurcation lesions which accounts for approximately 15 to 20% of patients undergoing this invasive procedure [1] has often been challenging for interventional cardiologists [2]. Even if the estimated annual cost of PCI for bifurcation lesions in the United States is approximately $ 4.4 billion [3], what matters at a later stage is the technique applied in order to prevent future complications [4]. With recent technological progress in interventional cardiology, application of the correct intra-procedural technique is vital to generate beneficial outcomes after coronary angioplasty.

At present, it is still not clear which stenting technique should be applied during PCI with drug eluting stents (DES) for coronary bifurcation lesions. Several trials including the coronary bifurcations: application of the crushing technique using sirolimus-eluting stents) [CACTUS] trial [5], the nordic stent technique study [6], the double kissing crush culotte stenting for the treatment of unprotected distal left main bifurcation lesions (DKCRUSH III) trial [7] have been set up to demonstrate the best intra-operative technique which should be applied for the treatment of bifurcation lesions during PCI.

Nevertheless, the post interventional cardiovascular outcomes associated with different techniques for bifurcation lesions have seldom been systematically studied.

As systematic reviews and meta-analyses might provide practitioners a vehicle to gain access to pre-filtered evidence and considerably save their time and expertise as well as shorten the knowledge gap in the literature to implement evidence-based practice, it was high time to systematically compare stenting techniques for bifurcation lesions.

In this analysis, we aimed to systematically compare the post interventional cardiovascular outcomes observed using the crush versus the provisional stenting techniques for bifurcation lesions.

## Methods

### Search databases and search strategies (including search terms)

Following the PRISMA guideline [8], a computerized search was carried out through medical literature analysis and retrieval system online (MEDLINE) and via its interface pubmed, through the biomedical and pharmacological bibliographic database excerpta medica database (EMBASE), through the Cochrane database and through www.ClinicalTrials.gov for English publications comparing crush versus the provisional stenting techniques for coronary bifurcation lesions.

The following search terms were used: “bifurcation lesions and percutaneous coronary intervention”, “bifurcation lesions and coronary angioplasty”, “bifurcation lesions and revascularization”, “crush stenting versus provisional stenting”, “crush stenting and percutaneous coronary intervention”, “crush stenting and PCI”, “crush stenting and provisional stenting and percutaneous coronary intervention”.

All the above mentioned databases were searched for relevant publications using these terms. Reference lists were also filtered for any relevant publication.

### Criteria for inclusion and exclusion

Inclusion criteria consisted of:

1. Studies (randomized trials and observational registries) which compared crush versus provisional stenting techniques in patients with coronary bifurcation lesions undergoing PCI;
2. Studies with the above criteria number (1) and additionally reporting adverse cardiovascular outcomes as their main endpoints.

Exclusion criteria consisted of:

1. Studies that compared crush versus culotte stenting techniques;
2. Studies that compared simple versus complex stenting without any specific precision of the type of stenting techniques which were used;
3. Studies that did not report adverse cardiovascular outcomes as their endpoints;
4. Studies that reported data which could not be used in this meta-analysis;
5. Studies that were literature reviews/meta-analyses/case studies/letters to editors;
6. Duplicated studies.

### Types of lesions, outcomes and follow-up time periods

All the participants were patients with coronary bifurcation lesions who were re-vascularized by PCI as shown in Table 1.

**Table 1:** Types of lesions, outcomes reported and follow-up time periods

Studies	Types of lesions + procedure	Outcomes reported	Follow-up time periods
Baystrukov2017 [9]	Bifurcation lesions + PCI	Cardiac death, MI, ST, TVR, stroke, MACE, re-occlusion	12 months
CACTUS [5]	True coronary bifurcation + PCI	MACE, MI, TLR, TVR, death	1 and 6 months
DKCRUSH II [10]	Coronary artery bifurcation lesions + PCI	MACE, cardiac death, MI, TLR, TVR, ST	60 months
DKCRUSH V [11]	Left main distal bifurcation lesions + PCI	Cardiac death, MI, TLR, ST, MACE, all-cause mortality, all revascularization	1 and 12 months
Galassi2009 [12]	Bifurcation lesions + PCI	MACE, TVR, TLR, MI, ST, Cardiac death, all-cause mortality	1 month and 24 months
Kim2015 [13]	Coronary artery bifurcation lesions with or without side branch + PCI	All-cause mortality, cardiac death, MI, TVR, TLR, ST, MACE	12 months

Abbreviations: *PCI* percutaneous coronary intervention, *MI* myocardial infarction, *ST* stent thrombosis, *TVR* target vessel revascularization, *TLR* target lesion revascularization, *MACE* major adverse cardiac events

The outcomes which were assessed included:

- Major adverse cardiac events (MACEs) consisting of the total number of death [cardiac and non-cardiac], myocardial infarction and revascularization [target vessel revascularization and/or target lesion revascularization] in combination;
- All-cause mortality;
- Cardiac death;
- Myocardial infarction (MI);
- Target vessel revascularization (TVR);
- Target lesion revascularization (TLR);
- Stent thrombosis (ST).

A minimum follow-up time period of 6 months and a maximum follow-up time period of 60 months were reported. Therefore, this analysis had a follow-up time period ranging from 6 to 60 months as shown in Table 1.

### Data extraction and quality assessment

Relevant data including the methodological features, the names of authors, year of publication, time period of patients’ enrollment, total number of participants assigned to the crush and provisional stenting groups, the follow-up time periods, the percentage of patients with diabetes mellitus, hypertension, dyslipidemia, current smoker, the relevant left ventricular ejection fraction, lesion length diameters for the main and side branches, mean age of the participants, the percentage of male and female patients, and the number of events reported for each outcome were independently extracted by two experts.

Any disagreement was carefully discussed and a final decision was made by the corresponding author.

The methodological assessment of the trials was carried out with strict reference to the recommendations by the Cochrane collaboration [14]. Grades were allotted to represent the risk of bias. A grade A denoted a low risk of bias, grade B denoted a moderate risk and a grade C denoted a high risk of bias.

### Statistical analysis

Statistical analysis was carried out by the most relevant RevMan 5.3 software. Odds ratios (OR) and 95% confidence intervals (CI) were generated during statistical analysis to represent the data.

The Q statistic test (where a *P* value of less or equal to 0.05 was considered statistically significant) and the I^2^ statistic test (a greater value denoting higher heterogeneity and a lower value denoting lower heterogeneity) were used to assess heterogeneity which was often observed in meta-analyses.

Either a fixed effect (I^2^ ≤ 50%) model or a random effect (I^2^ > 50%) model was used during statistical analysis depending upon the value of heterogeneity which was generated.

Sensitivity analysis was also carried out (by exclusion of each study by turn) to observe any significant difference from the main analytical results.

In addition, since this analysis comprised of a very small volume of studies, publication bias was visually assessed through funnel plots.

### Ethical approval

This is an analysis whereby data were collected from other published studies and therefore, ethical or institutional board review approval was not required.

## Results

### Search outcomes

Computerized literature search resulted in a total number of 1654 publications.

Following an initial assessment of the titles and the abstracts, several publications were rejected due to irrelevance and only 82 full text articles were assessed for eligibility.

Further assessment based on the inclusion and exclusion criteria resulted in another set of elimination of the full text articles:

- One (1) meta-analysis;
- Three (3) letters to editors;
- Five (5) case studies;
- Three (3) literature reviews;
- Trials reporting crush versus culotte stenting (6);
- Trials reporting provisional versus routine T stenting (3);
- Trials reporting simple versus complex stenting (3);
- Trials reporting tryton stent versus provisional stenting (1);
- Duplicated studies (51).

**Fig. 1:**
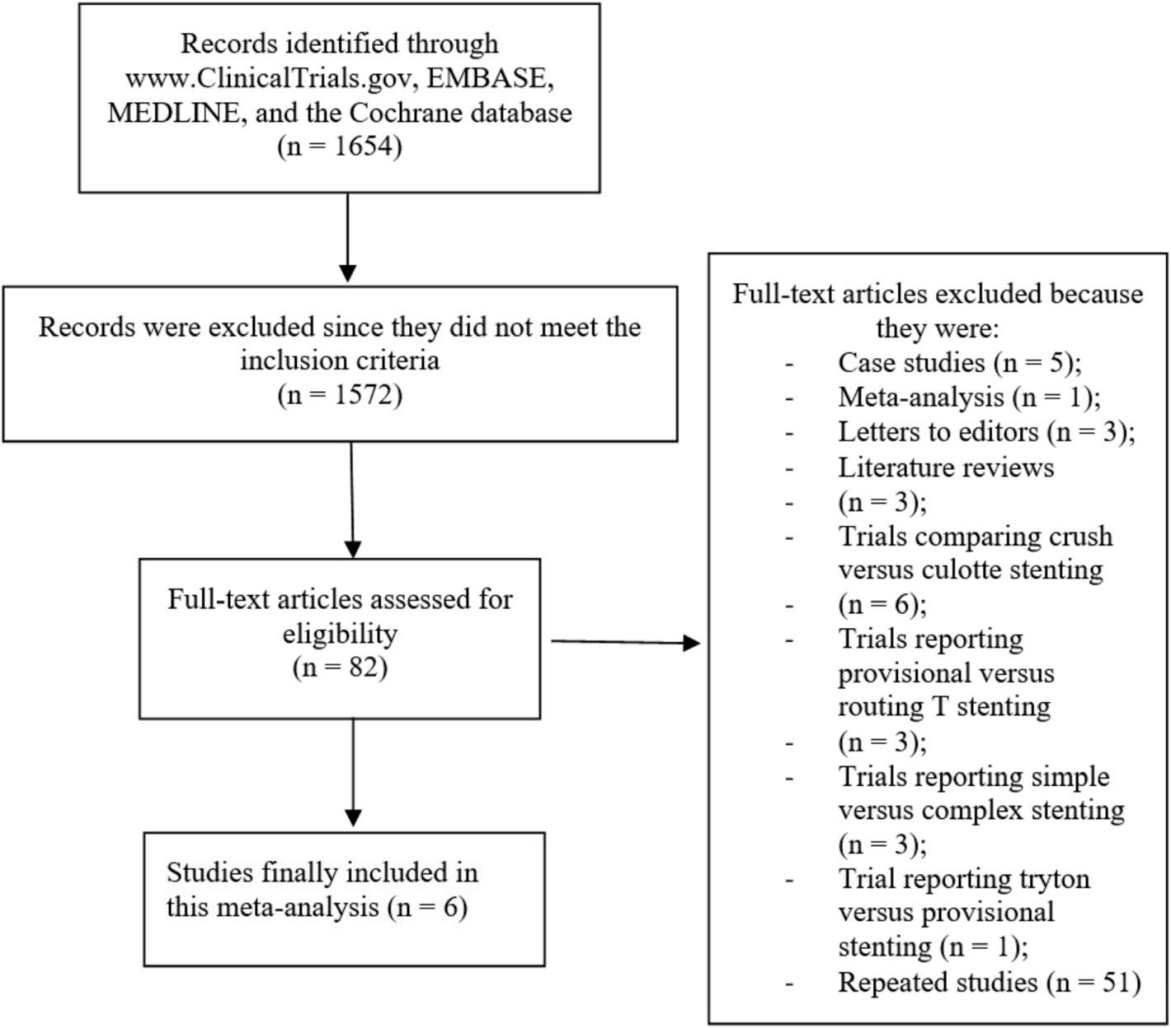
Flow diagram representing the study selection for crush versus provisional stenting technique during percutaneous coronary intervention for coronary bifurcation lesions

Finally only 6 studies [5, 9–13] were selected for this analysis as shown in Fig. 1.

### Main features of the relevant studies

Six studies with a total number of 2220 participants (1085 participants were assigned to the crush stenting technique and 1135 participants were assigned to the provisional stenting technique) were included in this analysis. The time period of patients’ enrollment was between years 2004 and 2016 as shown in Table 2.

**Table 2:** Main features of the studies

Studies	No of participants assigned to crush technique (n)	No of participants assigned to provisional stenting technique (n)	Time period of patients’ enrollment (years)	Type of study	Bias risk grade
Baystrukov2017	73	73	2011–2013	RCT	B
CACTUS	177	173	2004–2007	RCT	A
DKCRUSH II	183	183	2007–2009	RCT	A
DKCRUSH V	240	242	2011–2016	RCT	A
Galassi2009	199	258	2004–2006	OS	–
Kim2015	213	206	2008–2015	RCT	A
Total no of patients (n)	1085	1135			

Abbreviations: *RCT* randomized controlled trials, *OS* observational studies

Following the methodological assessment, four trials were graded into the “A category” implying a low risk of bias, and one trial was graded into the “B category” indicating a low to moderate risk of bias as shown in Table 2.

The baseline features of the participants have been listed in Table 3. Mean age was reported in years, and the other features were reported in percentage or millimeters.

**Table 3:** Baseline characteristics of the participants and lesions

Studies	Baystrukov2017	CACTUS	DKCRUSH II	DKCRUSH V	Galassi2009	Kim2015
Features	CT/PS	CT/PS	CT/PS	CT/PS	CT/PS	CT/PS
Mean Age (years)	57.3/58.5	65.0/67.0	63.9/64.7	65.0/64.0	62.2/64.5	60.9/61.1
Males (%)	75.3/78.1	80.2/76.3	78.8/75.8	82.9/77.7	83.9/73.4	75.1/75.2
Hypertension (%)	91.8/91.8	70.6/79.8	65.2/60.9	72.9/64.5	52.3/68.2	55.4/55.3
Diabetes mellitus (%)	24.7/24.7	23.7/22.0	19.6/23.1	28.8/25.6	30.7/33.5	25.8/29.1
Dyslipidemia (%)	63.0/60.3	63.8/70.5	33.7/29.1	47.5/47.5	60.8/57.3	62.0/57.3
Current smoker (%)	32.9/35.6	20.3/16.8	–	34.2/32.2	63.3/52.2	25.4/32.5
LVEF (%)	58.4/55.3	55.0/57.0	–	59.0/60.0	50.9/49.6	60.4/59.5
True BFL	67.1/64.4	100/100	–	–	–	–
SB diameter (mm)	2.30/2.40	–	–	–	2.55/2.54	–
Previous attempt (%)	2.70/8.20	–	–	–	–	–
Lesion length: main branch (mm)	–	–	25.8/25.8	27.9/28.8	–	28.9/27.8
Lesion length: side branch (mm)	–	–	15.3/14.6	21.0/21.3	–	10.3/8.30

Abbreviations: *CT* crush technique, *PS* provisional stenting, *SB* side branch, *LVEF* left ventricular ejection fraction, *BFL* bifurcation lesion, *mm* millimeters

### Main results of this analysis

**Fig. 2:**
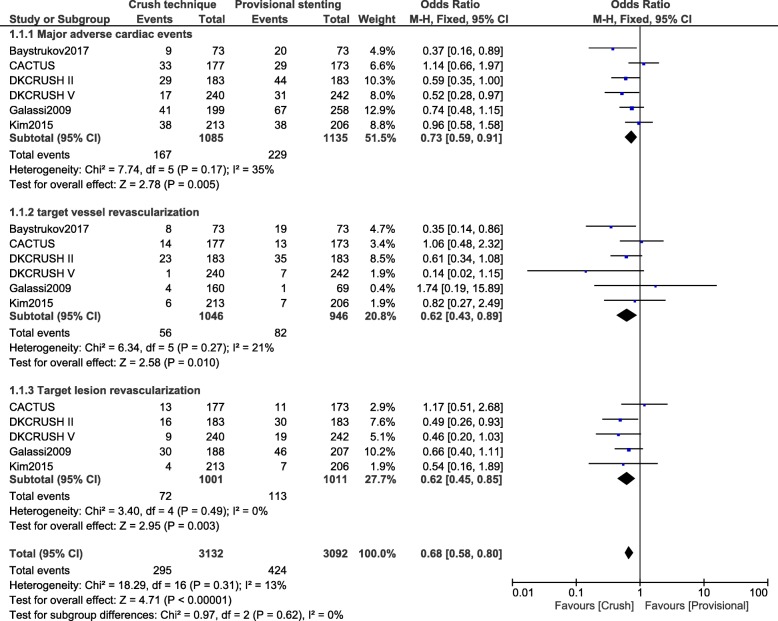
Cardiovascular outcomes observed between crush versus provisional stenting techniques following percutaneous coronary intervention for bifurcation lesions [1 month – 60 months] (part 1)

During a follow-up time period from six months to sixty months, crush stenting technique was associated with significantly lower major adverse cardiac events (OR: 0.73, 95% CI: 0.59–0.91; *P* = 0.005), TVR (OR: 0.62, 95% CI: 0.43–0.89; *P* = 0.01) and TLR (OR: 0.62, 95% CI: 0.45–0.85; *P* = 0.003) as compared to the provisional stenting technique for coronary bifurcation lesions (Fig. 2).

**Fig. 3:**
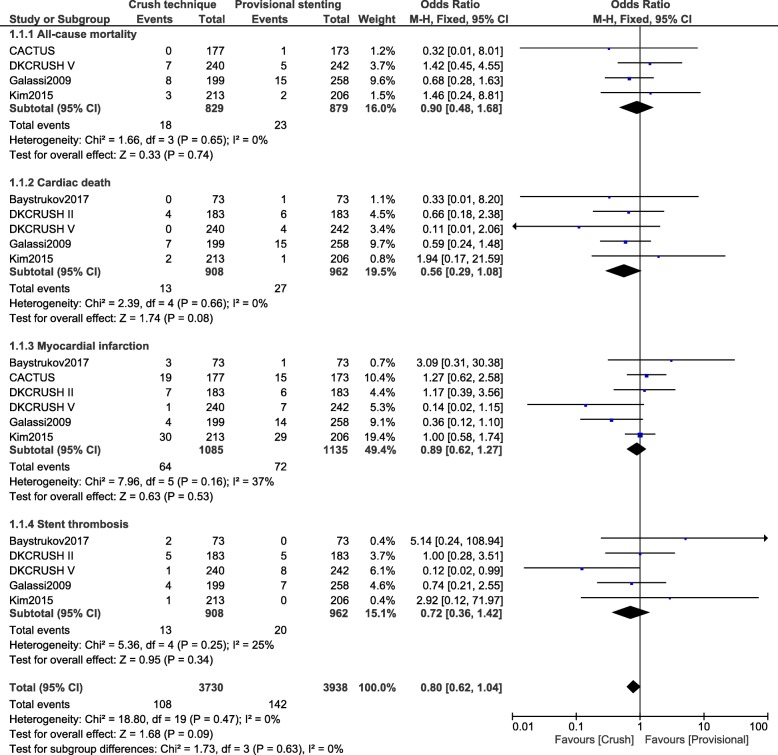
Cardiovascular outcomes observed between crush versus provisional stenting techniques following percutaneous coronary intervention for bifurcation lesions [1 month to 60 months] (part 2)

However, all-cause mortality (OR: 0.90, 95% CI: 0.48–1.68; *P* = 0.74), cardiac death (OR: 0.56, 95% CI: 0.29–1.08; *P* = 0.08), MI (OR: 0.89, 95% CI: 0.62–1.27; *P* = 0.53) and stent thrombosis (OR: 0.72, 95% CI: 0.36–1.42; *P* = 0.34) were not significantly different between the crush and the provisional stenting technique as shown in Fig. 3.

A summarized version of this main result has been given in Table 4.

**Table 4:** Results of this analysis

Outcomes assessed	Total no of studies involved (n)	OR with 95% CI	P value	I^2^ value (%)
Major adverse cardiac events	6	0.73 [0.59–0.91]	0.005	35
All-cause mortality	4	0.90 [0.48–1.68]	0.74	0
Cardiac death	5	0.56 [0.29–1.08]	0.08	0
Myocardial infarction	6	0.89 [0.62–1.27]	0.53	37
Target vessel revascularization	6	0.62 [0.43–0.89]	0.01	21
Target lesion revascularization	5	0.62 [0.45–0.85]	0.003	0
Stent thrombosis	5	0.72 [0.36–1.42]	0.34	25

Abbreviations: *OR* odds ratios, *CI* confidence intervals

**Fig. 4:**
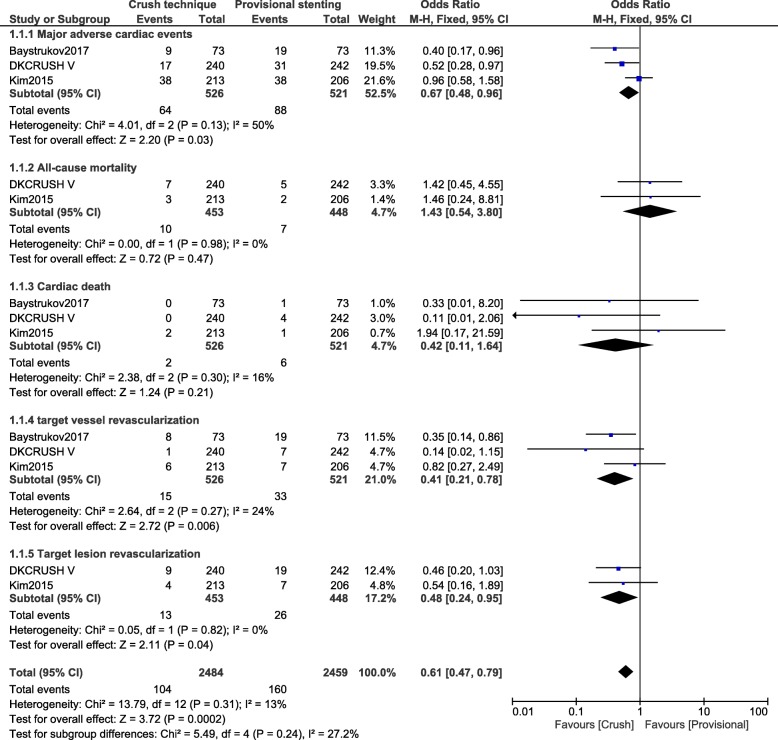
Cardiovascular outcomes observed between crush versus provisional stenting techniques following percutaneous coronary intervention for bifurcation lesions at 12 months (part 1)

**Fig. 5:**
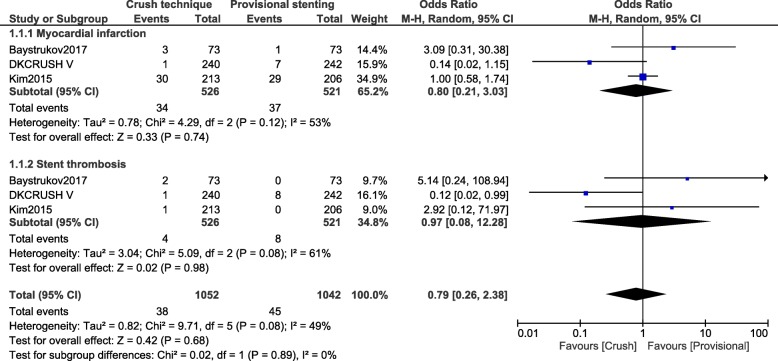
Cardiovascular outcomes observed between crush versus provisional stenting techniques following percutaneous coronary intervention for bifurcation lesions at 12 months (part 2)

Another separate analysis was carried out at 1 year follow-up. All the studies which reported outcomes at 12 months were included in this new analysis. Major adverse cardiac events (OR: 0.67, 95% CI: 0.48–0.96; *P* = 0.03), TVR (OR: 0.41, 95% CI: 0.21–0.78; *P* = 0.006) and TLR (OR: 0.48, 95% CI: 0.24–0.95; *P* = 0.04) still significantly favored the crush stenting technique as shown in Fig. 4. At 1 year follow-up, all-cause mortality (OR: 1.43, 95% CI: 0.54–3.80; *P* = 0.47), cardiac death (OR: 0.42, 95% CI: 0.11–1.64; *P* = 0.21), MI (OR: 0.80, 95% CI: 0.21–3.03; *P* = 0.74) and stent thrombosis (OR: 0.97, 95% CI: 0.08–12.28; *P* = 0.98) were similarly manifested between the crush versus the provisional stenting technique as shown in Figs. 4 and 5.

### Sensitivity analyses and publication bias

When an analysis was carried out without study Baystrukov2017, the results for important outcomes such as major adverse cardiac events (OR: 0.77, 95% CI: 0.61–0.97; *P* = 0.02), cardiac death (OR: 0.57, 95% CI: 0.29–1.12; *P* = 0.10), MI (OR: 0.86, 95% CI: 0.60–1.23; *P* = 0.41) and stent thrombosis (OR: 0.61, 95% CI: 0.29–1.26; *P* = 0.18) were not significantly different when compared to the main results of this analysis.

When study Galassi2009 was excluded, there was still no significant change in the results with reference to the main analysis: major adverse cardiac events (OR: 0.73, 95% CI: 0.57–0.95; P = 0.02), all-cause mortality (OR: 1.23, 95% CI: 0.49–3.07; *P* = 0.65), cardiac death (OR: 0.53, 95% CI: 0.21–1.34; P = 0.18), MI (OR: 1.01, 95% CI: 0.69–1.48; *P* = 0.95), TVR (OR: 0.60, 95% CI: 0.41–0.87; *P* = 0.007), TLR (OR: 0.60, 95% CI: 0.40–0.89; *P* = 0.01) and stent thrombosis (OR: 0.71, 95% CI: 0.31–1.61; P = 0.41).

Consistent results were maintained throughout when the other remaining studies were excluded.

**Fig. 6:**
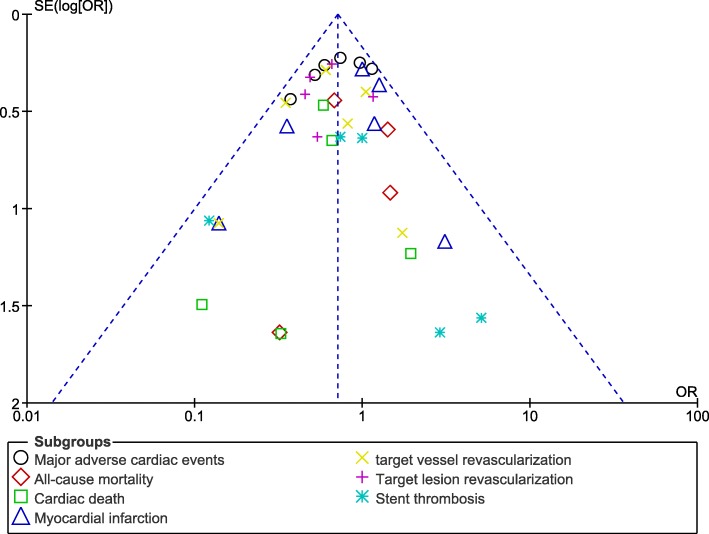
Funnel plot showing publication bias (A)

**Fig. 7:**
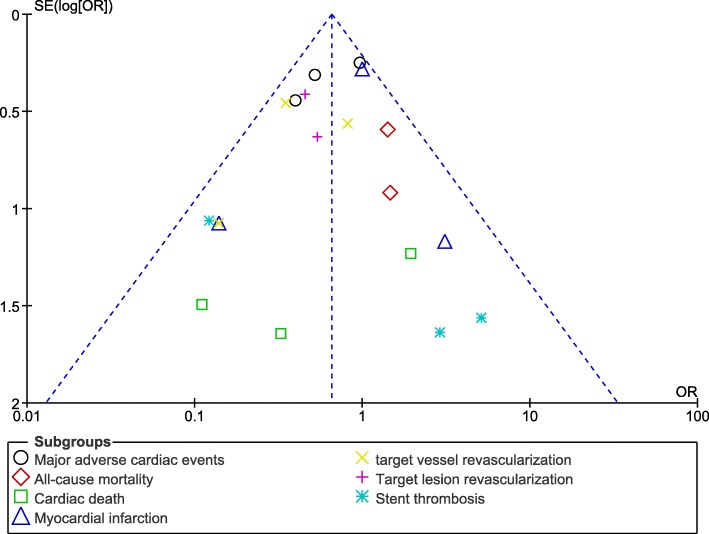
Funnel plot showing publication bias (B)

Publication bias, which was visually assessed through the funnel plots (Figs. 6 and 7), did not significantly vary (low evidence of publication bias) among all the studies that assessed the cardiovascular outcomes observed with the two different stenting techniques for coronary bifurcation lesions.

## Discussion

According to this current analysis, crush stenting technique was associated with significantly lower major adverse cardiac events, and significantly lower repeated revascularization rates compared to the provisional stenting technique for bifurcation lesions whereas mortality, MI and stent thrombosis were similarly manifested following this interventional procedure.

Similarly, results of the 3 year follow-up of the DKCRUSH-III study showed crush stenting to be associated with a significantly lower major adverse cardiac events as compared to the culotte stenting technique for unprotected left main distal bifurcation lesions [7]. And it was shown that major adverse cardiac events significantly increased with the culotte technique because of an increased rate of target vessel revascularization. This current analysis reported a significantly lower TVR and TLR with significantly reduced major adverse cardiac event associated with the crush stenting technique following the invasive procedure.

Results from the five year follow-up of the DKCRUSH-II (randomized study on double kissing crush technique versus provisional stenting technique for coronary artery bifurcation lesions) study showed crush stenting technique to be associated with a decreased rate of target lesion revascularization indicating an advantage of this stenting technique [10]. Our current analysis also showed the same result in terms of target vessel and target lesion revascularizations further confirming the results of the DKCRUSH-II trial.

In the CACTUS trial [5], the authors found that if stenosis was present in both branches of the bifurcation lesion, a provisional stenting of the main branch would be effective, but would also require the implantation of another stent on the side branch in one third of the number of patients. Major adverse cardiac events were similarly manifested in the crush stenting (15.8%) and the provisional stenting (15%) group. However, in this current analysis, which also included the CACTUS trial along with other relevant studies, major adverse cardiac events were significantly in favor of the crush stenting. The reason for such a result might have been the impact of revascularization (more number of events) as a major component of major adverse cardiac events.

Also, the DEFINITION Study (definitions and impact of complex bifurcation lesions on clinical outcomes after percutaneous coronary intervention using drug eluting stents) which compared provisional stenting and 2-stent strategies in patients with simple and complex bifurcation lesions showed both techniques to be almost similar in terms of major adverse cardiac events at 1 year follow-up [15].

Nevertheless, different from this current analysis, the TRYTON (prospective, single blind, randomized controlled study to evaluate the safety and effectiveness of the tryton side branch stent used with DES in treatment of de novo bifurcation lesions in the main branch and side branch in native coronaries) bifurcation Trial which randomly assigned 704 patients with bifurcation coronary lesions at 58 centers showed provisional stenting to remain the preferred strategy for non-left main true bifurcation lesions [16]. Also, a 5-year survival from patient-level pooled analysis of the nordic bifurcation study and the British bifurcation coronary study showed a provisional single stent approach to be associated with lower long term mortality in comparison to the dual stenting technique [17].

Few studies have also shown double stenting to be associated with higher major adverse cardiac events. Explanations might be related to the complexity of the appropriate lesions. More complicated coronary lesions might introduce higher complications during intra-procedural double stenting. The corresponding anatomical structure, torturous proximal, with moderate to severe calcification might further contribute to unwanted cardiac events post procedure [15]. Moreover, narrow bifurcation angle of ostial side branch might be rather challenging and might thus increase the changes for future stent thrombosis [18].

Other stenting techniques were associated with unwanted outcomes. Results from the British bifurcation coronary study: old, new, and evolving strategies showed that when coronary bifurcation lesions were treated, significantly increased major adverse cardiac events were observed with the systemic 2-stent technique and the reason was mainly due to MI reported during the procedure [19]. In addition, insight from in vitro experiments and micro-computed tomography showed crush technique to be associated with higher risks of mal-apposition than culotte or the T technique [20].

This systematic review and meta-analysis has briefly shown the post percutaneous coronary interventional outcomes observed in patients who were assigned to the crush versus the provisional intra-procedural stenting technique for bifurcation lesions. This evidence based analysis might be of some importance to interventional cardiologists.

### Limitations

This current meta-analysis has the following limitations: In general, the total number of participants who were assigned to the crush stenting versus the provisional stenting technique was limited. Therefore, our first limitation would be the insufficient total number of participants in comparison to other meta-analyses [21, 22]. However, the total number of participants were at least enough to reach a fair conclusion. Another limitation of this analysis would be the fact that antiplatelet therapies and other cardiac medications were not taken into consideration. In addition, the duration of antiplatelet treatment was also completely ignored, and this might have influenced the results. In this analysis, five studies were randomized controlled trials and one study was an observational study. As data extracted from observational studies were not as efficient in comparison to data which were extracted from randomized controlled trials, data from the observational cohort might have introduced heterogeneity and contributed to bias. Nevertheless, one study would not affect the final results as such. Also, the DEFINITION and the TRYTON studies could not be included in this analysis since they did not satisfy our inclusion criteria. Furthermore, in different studies, different types of drug eluting stents were used, and this could have had an impact on this analysis but we could not rectify this issue since the comparison of the two stenting techniques were not classified according to drug eluting stents. Also, even if a subgroup analysis based on left main versus non-left main bifurcation for PCI would have been of great interest, due to our limited data which did not sufficiently compare these two features, a comparison was not possible.

## Conclusion

In patients with coronary bifurcation lesions undergoing PCI, crush stenting technique was associated with significantly lower major adverse cardiac events and repeated revascularization without any change in mortality, myocardial infarction and stent thrombosis when compared to the provisional technique showing a benefit of crush stenting over the provisional stenting technique during PCI.

## Abbreviations

CBL: Coronary bifurcation lesions
MACEs: Major adverse cardiac events
OR: Odds ratios
PCI: Percutaneous coronary intervention
